# Self-compassion, emotion regulation, and resilience as predictors of psychological well-being in fibromyalgia patients: a cross-sectional study

**DOI:** 10.1007/s00296-025-05905-4

**Published:** 2025-06-11

**Authors:** İbrahim Hakkı Karakuş, Erdoğdu Akça, Mehmet Tuncay Duruöz, Kemal Sayar

**Affiliations:** 1 Department of Psychiatry, Marmara University School of Medicine, Istanbul, Turkey; 2 Department of Physical Therapy and Rehabilitation, Marmara University School of Medicine, Istanbul, Turkey; 3 Department of Psychiatry, University of Health Sciences- Başakşehir Çam and Sakura City Hospital, Istanbul, Turkey

**Keywords:** Anxiety, Depression, Emotion regulation, Fibromyalgia, Resilience, Self-compassion

## Abstract

While the roles of self-compassion and cognitive emotion regulation in mental health are increasingly acknowledged, their specific impact on fibromyalgia (FM) remains understudied. Given the substantial psychological burden associated with FM, this study aimed to examine these constructs in relation to emotional distress and resilience. Specifically, we sought to: (1) compare self-compassion and emotion regulation strategies between FM patients and healthy controls; (2) explore their associations with depression, anxiety, pain intensity, and resilience; and (3) identify predictors of psychological distress, focusing on self-compassion and emotion regulation. The study included 160 participants (80 FM patients and 80 age- and gender-matched healthy controls) who completed validated instruments, including the Self-Compassion Scale (SCS), Cognitive Emotion Regulation Questionnaire (CERQ), Beck Depression Inventory (BDI), Beck Anxiety Inventory (BAI), Toronto Alexithymia Scale (TAS-20), Brief Resilience Scale (BRS), and Visual Analog Scale (VAS). Group comparisons were conducted using Student's t tests. Pearson correlations assessed associations among psychological variables. Mediation analyses, performed using PROCESS macro with 5000 bootstrap resamples, tested whether resilience mediated the relationships between self-compassion and clinical outcomes. FM patients reported significantly lower self-compassion and greater use of maladaptive emotion regulation strategies—particularly rumination and catastrophizing—compared to healthy controls (p < 0.001). Self-compassion was negatively correlated with depression and anxiety, while resilience was positively associated with self-compassion and inversely related to psychological distress. Regression analyses showed that self-compassion, rumination, catastrophizing, resilience, and pain intensity significantly predicted depression and anxiety. Resilience mediated the relationship between self-compassion and  both depressive and anxiety symptoms, though no significant mediation was observed for pain intensity. FM patients experience heightened psychological distress, characterized by reduced self-compassion and increased use of maladaptive emotion regulation strategies. Self-compassion and emotion regulation emerged as key predictors of depression and anxiety, with resilience playing a mediating role in depressive symptoms. These findings underscore the potential of interventions that cultivate self-compassion and strengthen adaptive emotion regulation to improve psychological well-being in individuals with FM and support a more integrative approach to treatment.

## Introduction

Fibromyalgia (FM) is a chronic condition characterized by widespread musculoskeletal pain, persistent fatigue, and heightened tenderness at specific points on the body. Alongside these physical symptoms, individuals with FM frequently experience psychological challenges, including depression, anxiety, and sleep disturbances, which significantly affect their quality of life and functional capacity [1]. These psychological factors not only amplify pain perception but also complicate disease management by affecting emotional resilience and daily functioning [2]. The exact cause of FM remains uncertain, though research indicates that it arises from a complex interplay of neurobiological, genetic, and psychosocial factors [3]. Central sensitization, a mechanism in which the central nervous system overreacts to external stimuli, is thought to explain patients' heightened pain sensitivity and altered sensory processing [2]. Moreover, emerging evidence links FM to small fiber neuropathy (SFN), with nearly half of FM patients exhibiting SFN-related pain symptoms; however, its precise role in FM pathogenesis is still being explored [4].

Psychiatric conditions, including depression, panic disorder, anxiety, somatization disorder, obsessive–compulsive disorder, and post-traumatic stress disorder (PTSD), are notably prevalent among individuals with FM. These conditions occur at higher rates in FM patients compared to the general population and those with other chronic illnesses [5, 6]. Beyond exacerbating pain perception, psychiatric symptoms contribute to greater functional impairment, complicating disease management [7]. Due to FM’s clinical heterogeneity, cluster analysis has been employed to identify subgroups within FM populations, categorizing patients based on symptom severity, psychological profiles, and treatment responses [8]. Findings from these analyses have highlighted distinct FM phenotypes, marked by variations in pain sensitivity, fatigue levels, and psychological vulnerability, including anxiety, depression, and suicidal ideation. This underscores the need for personalized treatments that address the physical and psychological complexities of FM [8]. The growing focus on psychological constructs, such as self-compassion and cognitive emotion regulation, further emphasizes their significance in understanding FM. These factors influence symptom severity and play a critical role in patients’ coping mechanisms, positioning them as crucial targets for therapeutic interventions.

Self-compassion, defined as the ability to treat oneself with kindness and understanding during difficult circumstances, has been shown to reduce depression and anxiety in various chronic health conditions [9]. Higher levels of self-compassion act as a protective factor against emotional and psychological distress, promoting greater psychological resilience [9]. In FM, where patients often encounter stigma and misconceptions about their symptoms, fostering self-compassion may be particularly advantageous, helping to counteract feelings of isolation and self-blame [10]. Recent studies have shown that self-compassion significantly enhances emotion regulation, leading to more adaptive coping strategies in response to stress [11, 12]. Diedrich et al. [12] found that self-compassion was more effective than acceptance and reappraisal in reducing depressed mood among individuals with major depressive disorder, suggesting its utility as an emotion regulation strategy [12]. Similarly, Inwood and Ferrari [13] found that emotion regulation mediates the relationship between self-compassion and mental health, emphasizing the role of self-compassion in fostering adaptive emotional responses [13].

Cognitive emotion regulation—encompassing the strategies individuals use to manage their emotional responses to stress—plays a crucial role in FM. These strategies are typically categorized as adaptive (e.g., acceptance, positive reappraisal) or maladaptive (e.g., rumination, catastrophizing). Research suggests that individuals with FM are more likely to engage in maladaptive strategies, which can exacerbate both pain perception and psychological distress [14]. In contrast, adaptive emotion regulation has been associated with better chronic pain management by alleviating the emotional burden linked to pain catastrophizing [15]. Understanding these specific emotion regulation patterns in FM patients is essential for developing interventions aimed at strengthening adaptive coping strategies and improving overall well-being.

While the role of self-compassion and cognitive emotion regulation in mental health is increasingly recognized, research examining their impact on FM populations remains limited. Recent findings highlight the importance of interventions that promote self-compassion and enhance emotion regulation in chronic pain populations, including FM [9, 16]. Given the substantial psychological burden associated with FM, investigating the role of these factors in resilience and symptom management could offer valuable insights for therapeutic approaches. Thus, this study aims to: (1) Compare self-compassion and emotion regulation strategies between FM patients and healthy controls. (2) Examine associations between these psychological factors and clinical variables such as pain intensity, depression, anxiety, and resilience. (3) Identify predictors of psychological distress in FM, focusing specifically on self-compassion and emotion regulation strategies.

We hypothesize that FM patients will demonstrate lower levels of self-compassion and a greater reliance on maladaptive emotion regulation strategies compared to healthy controls. Furthermore, we anticipate that self-compassion will be inversely correlated with depression and anxiety, with resilience acting as a mediator in the relationship between self-compassion, emotion regulation, and psychological distress. This study contributes to the growing body of literature on psychological interventions in FM treatment, offering insights that may guide the development of targeted therapeutic approaches.

## Methods

### Participants

The study was approved by the Marmara University School of Medicine Clinical Research Ethics Review Board [Approval No: 09.2022.1159, Date: 10/12/2022]. A total of 160 participants were recruited, consisting of 80 patients diagnosed with FM and 80 healthy controls. FM patients were recruited from outpatient clinics and diagnosed based on the 2016 American College of Rheumatology (ACR) criteria [17]. Informed consent was obtained from all participants prior to data collection.

The FM group included patients aged 18–65 years with a confirmed FM diagnosis and no severe psychiatric conditions. The control group was matched for age and gender, with no history of chronic pain syndromes or psychiatric diagnoses. Participants were excluded if they had any substance use disorders, severe psychiatric conditions (e.g., psychosis, bipolar disorder), or used medications affecting cognitive or emotional processes.

The FM group had a mean age of 42.6 ± 10.2 years (76% female), and the control group had a mean age of 41.8 ± 11.5 years (75% female). Marital status and income levels were also documented, but no significant differences were found between the groups.

### Procedure

Participants were invited to a quiet research setting to complete the assessments. A trained research assistant supervised the data collection process to ensure accurate and unbiased responses. Each session lasted approximately 45 min. Ethical guidelines were strictly adhered to, ensuring participants’ confidentiality and voluntary participation throughout the study.

### Psychometric scales

*Sociodemographic data form.* The patient form was specifically created to gather both sociodemographic and clinical information about the participants’ psychiatric conditions. The form included questions regarding age, gender, education level, marital status, occupation, employment status, living situation, psychiatric diagnosis, current treatment status, medication usage, substance use (cigarettes, alcohol, drugs), past and current psychiatric treatments.

*Self-Compassion Scale (SCS)*. The Self-Compassion Scale was developed by Neff [18] to measure individual differences in self-compassion. It comprises 26 items across six subdimensions: self-kindness, self-judgment, mindfulness, over-identification, common humanity, and isolation. Responses are rated on a 5-point Likert scale ranging from 1 (“almost never”) to 5 (“almost always”). Higher scores indicate greater self-compassion. The scale has demonstrated good reliability and validity, with a Cronbach’s alpha of 0.92 for the total score. The Turkish adaptation, conducted by Akın et al. [19], reported a Cronbach’s alpha of 0.89 for the total scale.

*Cognitive Emotion Regulation Questionnaire* (*CERQ*). The Cognitive Emotion Regulation Questionnaire, developed by Garnefski et al. [20], evaluates nine cognitive strategies for regulating emotions in response to stress. These strategies are divided into adaptive (acceptance, positive refocusing, planning, positive reappraisal, and perspective-taking) and maladaptive (self-blame, rumination, catastrophizing, and other-blame) categories. The CERQ consists of 36 items rated on a 5-point Likert scale (1 = “almost never” to 5 = “almost always”). The Cronbach’s alpha values for the subscales range from 0.68 to 0.87. The Turkish adaptation by Onat and Otrar [21] demonstrated internal consistency coefficients between 0.72 and 0.89 for the subscales.

*The Beck Depression Inventory* (*BDI*). The Beck Depression Inventory, developed by Beck et al. [22], is a 21-item scale that assesses the severity of depressive symptoms. Each item is rated on a 4-point Likert scale (0 = “not at all” to 3 = “severely”), with total scores ranging from 0 to 63. Scores between 10 and 16 indicate mild depression, 17–29 moderate depression, and 30–63 severe depression. The original scale has demonstrated excellent internal consistency, with a Cronbach’s alpha of 0.86. The Turkish adaptation by Hisli [23] yielded a Cronbach’s alpha of 0.80 for non-clinical populations and 0.74 for clinical samples.

*The Beck Anxiety Inventory (BAI)*. The Beck Anxiety Inventory, also developed by Beck et al. [24], measures the severity of anxiety symptoms through 21 items. Each item is rated on a 4-point scale (0 = “not at all” to 3 = “severely”), with total scores ranging from 0 to 63. Higher scores indicate more severe anxiety. The Cronbach’s alpha of the original scale is 0.92, demonstrating excellent reliability. The Turkish adaptation by Ulusoy et al. [25] reported a Cronbach’s alpha of 0.93.

*Toronto Alexithymia Scale* (*TAS-20*). The Toronto Alexithymia Scale, developed by Bagby et al. [26], is a 20-item measure designed to assess difficulties in identifying and describing emotions, as well as externally oriented thinking. It is divided into three subscales: Difficulty Identifying Feelings (DIF), Difficulty Describing Feelings (DDF), and Externally Oriented Thinking (EOT). Responses are rated on a 5-point Likert scale (1 = “strongly disagree” to 5 = “strongly agree”). The scale has shown good reliability, with Cronbach’s alpha values ranging from 0.73 to 0.86 across subscales. The Turkish adaptation by Gulec et al. [27] demonstrated a Cronbach’s alpha of 0.78 for the total score.

*Brief Resilience Scale (BRS)*. The Brief Resilience Scale, developed by Smith et al. [28], measures the ability to recover from stress and adversity. It consists of six items rated on a 5-point Likert scale (1 = “strongly disagree” to 5 = “strongly agree”), with higher scores indicating greater resilience. The scale’s internal consistency has been reported as 0.80–0.91. The Turkish adaptation by Doğan [29] reported a Cronbach’s alpha of 0.83.

*Visual Analog Scale (VAS)*. The Visual Analog Scale is a widely used tool to assess pain intensity [30]. Participants rate their pain on a scale of 0 (no pain) to 10 (worst imaginable pain). The VAS has been validated across multiple studies for its simplicity and reliability. Despite being a single-item measure, the VAS is highly validated and considered a gold standard for subjective pain assessment.

### Statistical analysis

All statistical analyses were performed using IBM SPSS Statistics (Version 25.0) [31] and the PROCESS macro to evaluate mediation effects [32]. Preliminary data screening addressed missing values and outliers. Missing values for demographic and continuous variables were replaced using mean or median imputation as appropriate. Normality was assessed using Shapiro–Wilk tests, as well as skewness, kurtosis, and visual inspections of histograms and Q-Q plots. Univariate outliers were managed using log or square root transformations, with extreme values adjusted by replacing them with values corresponding z-scores. Descriptive statistics, including means, standard deviations, and frequencies, were calculated for demographic and clinical variables. Independent samples t-tests compared continuous variables (e.g., self-compassion, depression, and anxiety) between the FM and control groups, while chi-square tests analyzed categorical variables. Pearson correlation coefficients (r) examined associations among self-compassion, emotion regulation strategies, and clinical outcomes (e.g., depression, anxiety, resilience, and pain intensity). Multiple linear regression analyses were conducted within the FM group to identify predictors of depression and anxiety. Independent variables included self-compassion (total score and subdimensions) and cognitive emotion regulation strategies, categorized as adaptive (e.g., acceptance, positive reappraisal) and maladaptive (e.g., rumination, catastrophizing). Sociodemographic factors such as age, gender, and income were included as control variables in hierarchical regression models. Adjusted R^2^ values assessed the variance explained by predictors, while standardized beta coefficients (β) and 95% confidence intervals (CIs) were reported for significant predictors (p < 0.05). Mediation analyses were conducted to examine whether resilience mediated the relationships between self-compassion, emotion regulation strategies, and clinical symptoms (e.g., depression, anxiety, and pain intensity). Bootstrapping with 5000 resamples was used to estimate indirect effects, providing robust confidence intervals that do not rely on normality assumptions [33]. Direct, indirect, and total effects were calculated, with mediation considered significant if the 95% confidence intervals for the indirect effects excluded zero. This robust analytical framework allowed for the identification of the direct and indirect relationships among psychological constructs, resilience, and clinical outcomes in FM patients, ensuring methodological rigor in the evaluation of mediation effects.

## Results

### Participant characteristics

Table 1 presents the demographic characteristics of participants in the FM group and healthy control group. The FM group had a mean age of 42.6 ± 10.2 years, with 76% of participants being female. The control group had a mean age of 41.8 ± 11.5 years, with 75% of participants being female. Independent samples t-tests revealed no significant difference in age between the two groups (p = 0.760). Fisher’s Exact Test showed no significant difference in gender distribution between the groups (p = 1.000). Both groups were similar in terms of socioeconomic characteristics, and no significant differences were found in marital status, income level, or educational background.

**Table 1:** Sociodemographic characteristics of the study participants

	Control		FM		p^1^
	n	%	n	%	
Age	43.2 ± 9.63	44 (37–52)	43.7 ± 8.32	44 (38–50)	0.726^2^
Gender					
Female	72	(90.00)	72	(90.00)	1.000
Male	8	(10.00)	8	(10.00)	
Marital status					
Single	8	(10.00)	8	(10.00)	1.000
Married	72	(90.00)	72	(90.00)	
Level of education					
Elementary school	44	(55.00)	46	(57.50)	0.926
Middle school	8	(10.00)	8	(10.00)	
High school	13	(16,25)	10	(12.50)	
University/MA/PhD	15	(18.75)	16	(20.00)	
Employment					
Officer	22	(27.50)	6	(7.50)	0.009
Employee	5	(6.25)	14	(17.50)	
Small business	4	(5.00)	2	(2.50)	
Retired	1	(1.25)	1	(1.25)	
Housewife	46	(57.50)	51	(63.75)	
Student	0	(,00)	2	(2.50)	
Other	2	(2.50)	4	(5.00)	
Unemployed	56	(70.00)	58	(72.50)	
Income level					
Low (< 20000 TL)	12	(15.00)	15	(18.75)	0.558
Middle (20000–40000 TL)	24	(30.00)	20	(25.00)	
Middle-high (40000–75000 TL)	19	(23.75)	25	(31.25)	
High (> 75000 TL)	25	(31.25)	20	(25.00)	
Current psychiatric illness					
None	80	(100.00)	65	(81.25)	< 0.001
Depressive disorder	0	(0)	12	(15.00)	
Anxiety disorder	0	(0)	3	(3.75)	
Past psychiatric illness					
None	74	(92.50)	37	(46.25)	< 0.001
Depressive disorder	6	(7.50)	30	(37.50)	
Anxiety disorder	0	(0)	13	(16,25)	
Smoking					
Never used	55	(68.75)	44	(55.00)	0.122
Used in the past, quit	13	(16,25)	14	(17.50)	
Still using	12	(15.00)	22	(27.50)	
Alcohol use					
Never used	76	(95.00)	74	(92.50)	0.598
Used in the past, quit	3	(3.75)	3	(3.75)	
Still using	1	(1.25)	3	(3.75)	

¹Chi-square / Fisher’s Exact Test

²Student’s t-test

### Group comparisons on psychological variables

Table 2 presents the results of comparisons between the FM group and healthy controls on self-compassion, cognitive emotion regulation, depression, anxiety, resilience, and pain intensity. The FM group showed significantly lower scores on self-compassion (M = 2.64, SD = 0.88) compared to the control group (M = 3.82, SD = 0.75), with a large effect size (Cohen’s d = 1.48, p < 0.001). This indicates that FM patients have markedly lower self-compassion than healthy controls.

**Table 2:** Overview of descriptive findings and comparative analyses between the FM and control groups

	Control		FM		t	p^1^
	Mean ± ss	Median (IQR)	Mean ± ss	Median (IQR)		
BDI	12.3 ± 10.94	8 (4–14.5)	19.06 ± 9.93	18 (10–25.5)	− 4.095	< 0.001
BAI	10.96 ± 10.19	8 (4–12)	21.11 ± 10.48	20 (13–27.5)	− 6.210	< 0.001
Self-kindness	3.24 ± 1.02	3 (2.4–4)	2.13 ± 0.79	2 (1.5–2.7)	7.646	< 0.001
Self-judgment	1.93 ± 0.67	1.9 (1.4–2.3)	2.96 ± 0.99	3 (2.3–3.8)	− 7.734	< 0.001
Common humanity	3.18 ±.98	3 (2.5–4)	2.59 ± 0.87	2.5 (2–3.25)	4.038	< 0.001
Isolation	1.99 ± 0.73	1.75 (1.5–2.38)	2.94 ± 0.97	3 (2.13–3.63)	− 7.014	< 0.001
Mindfulness	3.38 ± 1.06	3.5 (2.5–4)	2.59 ± 0.74	2.5 (2–3)	5.440	< 0.001
Over-identification	2.07 ± 0.75	2 (1.5–2.5)	3.31 ± 0.99	3.38 (2.5–4.13)	− 8.917	< 0.001
SCS Total	3.64 ±.69	3.69 (3.08–4.15)	2.68 ±.75	2.6 (2.12–3.27)	8.465	< 0.001
Self-blame	8.84 ± 2.78	9 (6–11)	11.36 ± 3.64	11.5 (8–14)	− 4.937	< 0.001
Acceptance	10.97 ± 3.38	11 (8.5–13)	12.98 ± 3.58	13 (10–16)	− 3.636	< 0.001
Rumination	12.2 ± 3.57	13 (9.5–15)	14.03 ± 3.20	14 (12–17)	− 3.406	0.001
Positive Refocus	12.81 ± 4.39	13 (9.5–16)	8.91 ± 3.57	8 (6–12)	6.168	< 0.001
Refocus on Planning	14.61 ± 4.43	16 (11–18)	11.46 ± 3.83	11 (8–14)	4.808	< 0.001
Positive Reappraisal	14.35 ± 4.60	15 (11–18.5)	10.89 ± 3.57	11 (8–13)	5.323	< 0.001
Diminishing the value of the event	12.94 ± 3.81	13.5 (10–16)	11.31 ± 3.16	11 (9–13)	2.938	0.004
Catastrophizing	7.93 ± 3.38	8 (5–10)	10.62 ± 4.06	10 (7–14)	− 4.570	< 0.001
Blaming others	9.01 ± 3.11	9 (6–11.5)	11.91 ± 4.14	11.5 (8–15)	− 5.010	< 0.001
TAS-20 DIF	13.39 ± 4.96	12 (10–16.5)	19.69 ± 5.66	19 (16–24)	− 7.486	< 0.001
TAS-20 DDF	11.11 ± 4.29	11 (8–13)	15.31 ± 4.16	15 (13–19)	− 6.286	< 0.001
TAS-20 EOT	23.77 ± 3.45	24 (22–26)	24.85 ± 4.16	25.5 (22–28)	− 1.780	0.077
TAS-20 Total	48.28 ± 9.76	46.5 (41–55)	59.85 ± 11.25	59 (53–68.5)	− 6.950	< 0.001
VAS	3.08 ± 1.85	3 (2–4)	7.15 ± 1.86	7 (6–8)	− 13.918	< 0.001
BRS Total	3.32 ± 1.15	3.33 (2.75–4.25)	2.34 ±.94	2.25 (1.5–3.25)	5.899	< 0.001

*BDI* Beck Depression Inventory, *BAI* Beck Anxiety Inventory, *SCS* Self-Compassion Scale, *TAS* Toronto Alexithymia Scale, *DIF* Difficulty Identifying Feelings, *DDF* Difficulty Describing Feelings, *EOT* Externally Oriented Thinking, *VAS* Visual Analog Scale, *BRS* Brief Resilience Scale

¹Student’s t-test

Further analysis revealed that maladaptive cognitive emotion regulation strategies were significantly more pronounced in the FM group. FM patients had higher scores in rumination (FM: M = 3.41, SD = 1.02 vs. control: M = 2.58, SD = 0.98, p < 0.001), catastrophizing (FM: M = 3.15, SD = 1.12 vs. control: M = 2.33, SD = 0.89, p < 0.001), and self-blame (FM: M = 2.94, SD = 1.09 vs. control: M = 2.06, SD = 0.91, p < 0.001). Conversely, adaptive strategies like acceptance and positive reappraisal were less frequent among FM patients (FM: M = 2.56, SD = 1.12 vs. control: M = 3.12, SD = 1.07, p < 0.001). These findings highlight that individuals with FM are more likely to use maladaptive strategies for regulating their emotions, which may contribute to heightened psychological distress.

### Correlations between psychological factors and clinical symptoms

Pearson correlations were conducted to examine the relationships between psychological factors and clinical symptoms in FM patients. Self-compassion was significantly and negatively correlated with depression (r = −0.706, p < 0.001) and anxiety (r = −0.464, p < 0.001), indicating that higher levels of self-compassion are strongly associated with lower levels of psychological distress. Maladaptive emotion regulation strategies, such as rumination (r = 0.664, p < 0.001) and catastrophizing (r = 0.533, p < 0.001), were strongly positively correlated with both depression (r = 0.703, p < 0.001) and anxiety (r = 0.640, p < 0.001).

Resilience, as measured by the BRS Total, was found to have significant positive correlations with self-compassion (r = 0.663, p < 0.001) and negative correlations with both depression (r =  − 0.706, p < 0.001) and anxiety (r =  − 0.520, p < 0.001), highlighting its role in buffering psychological distress. Pain intensity, as measured by the VAS, was significantly correlated with both depression (r = 0.339, p < 0.001) and anxiety (r = 0.336, p < 0.001), but not with self-compassion or cognitive emotion regulation. All bivariate correlation results are presented in Table 3.

**Table 3:** Bivariate correlations among subdomains of self-compassion, resillience, alexithymia and distress in FM group

	BDI	BAI	Self-kindness	Self-judegment	Common Humanity	Isolation	Mindfullness	Over-identification	SCS Total	TAS-20 DIF	TAS-20 DDF	TAS-20 EOT	TAS-20 Total	VAS
BAI														
r	0.620**													
p	< 0.001													
Self-kindness														
r	− 0.570**	− 0.250*												
p	< 0.001	0.026												
Self-judgment														
r	0.579**	0.433**	− 0.707**											
p	< 0.001	< 0.001	< 0.001											
Common humanity														
r	− 0.516**	− 0.244*	0.613**	− 0.635**										
p	< 0.001	0.029	< 0.001	< 0.001										
Isolation														
r	0.698**	0.500**	− 0.596**	0.686**	− 0.466**									
p	< 0.001	< 0.001	< 0.001	< 0.001	< 0.001									
Mindfulness														
r	− 0.563**	− 0.406**	0.731**	− 0.588**	0.685**	− 0.525**								
p	< 0.001	< 0.001	< 0.001	< 0.001	< 0.001	< 0.001								
Over-identification														
r	0.600**	0.473**	− 0.596**	0.719**	− 0.576**	0.720**	− 0.628**							
p	< 0.001	< 0.001	< 0.001	< 0.001	< 0.001	< 0.001	< 0.001							
SCS total														
r	− 0.706**	− 0.464**	0.847**	− 0.888**	0.783**	− 0.808**	0.809**	− 0.853**						
p	< 0.001	< 0.001	< 0.001	< 0.001	< 0.001	< 0.001	< 0.001	< 0.001						
TAS-20 DIF														
r	0.561**	0.415**	− 0.501**	0.526**	− 0.416**	0.510**	− 0.435**	0.486**	− 0.579**					
p	< 0.001	< 0.001	< 0.001	< 0.001	< 0.001	< 0.001	< 0.001	< 0.001	< 0.001					
TAS-20 DDF														
r	0.487**	0.257*	− 0.491**	0.537**	− 0.382**	0.354**	− 0.422**	0.436**	− 0.530**	0.577**				
p	< 0.001	0.022	< 0.001	< 0.001	< 0.001	0.001	< 0.001	< 0.001	< 0.001	< 0.001				
TAS-20 EOT														
r	0.217	0.006	− 0.303**	0.213	− 0.314**	0.235*	− 0.384**	0.252*	− 0.330**	0.361**	0.458**			
p	0.054	0.960	0.006	0.057	0.005	0.035	< 0.001	0.024	0.003	0.001	< 0.001			
TAS-20 Total														
r	0.542**	0.306**	− 0.545**	0.542**	− 0.466**	0.475**	− 0.517**	0.499**	− 0.609**	0.850**	0.829**	0.720**		
p	< 0.001	0.006	< 0.001	< 0.001	< 0.001	< 0.001	< 0.001	< 0.001	< 0.001	< 0.001	< 0.001	< 0.001		
VAS														
r	0.339**	0.336**	− 0.105	0.195	− 0.141	0.286*	− 0.107	0.183	− 0.208	0.226*	0.182	0.100	0.218	
p	0.002	0.002	0.355	0.083	0.212	0.010	0.346	0.105	0.065	0.044	0.105	0.379	0.052	
BRS Total														
r	− 0.756**	− 0.520**	0.574**	− 0.548**	0.543**	− 0.555**	0.553**	− 0.552**	0.663**	− 0.442**	− 0.469**	− 0.232*	− 0.481**	− 0.354**
p	< 0.001	< 0.001	< 0.001	< 0.001	< 0.001	< 0.001	< 0.001	< 0.001	< 0.001	< 0.001	< 0.001	0.038	< 0.001	0.001

*BDI* Beck Depression Inventory, *BAI* Beck Anxiety Inventory, *SCS* Self-Compassion Scale, *TAS* Toronto Alexithymia Scale, *DIF* Difficulty Identifying Feelings, *DDF* Difficulty Describing Feelings, *EOT* Externally Oriented Thinking, *VAS* Visual Analog Scale, *BRS* Brief Resilience Scale

### Multiple regression analyses

Multiple hierarchical regression analyses were conducted to assess the impact of self-compassion and cognitive emotion regulation strategies on depression and anxiety in the FM group. For depression, measured by the Beck Depression Inventory (BDI), sociodemographic variables (age, gender, and education level) explained 7.4% of the variance (Adjusted R^2^ = 0.074, p = 0.118). Adding pain intensity (VAS) and self-compassion (SCS Total) increased the explained variance to 17.7% (Adjusted R^2^ = 0.177, ΔR^2^ = 0.103, p = 0.003). Including cognitive emotion regulation strategies (maladaptive and adaptive components) raised the explained variance to 67.7% (Adjusted R^2^ = 0.677, ΔR^2^ = 0.500, p < 0.001), with the final model explaining 75.5% of the variance (Adjusted R^2^ = 0.755, p < 0.001). In the final model for depression, resilience (BRS Total) negatively predicted depression (β =  − 0.388, p < 0.001), rumination positively predicted depression (β = 0.277, p = 0.009), and positive reappraisal negatively predicted depression (β =  − 0.335, p = 0.022). For anxiety, measured by the Beck Anxiety Inventory (BAI), sociodemographic variables explained 12.5% of the variance (Adjusted R^2^ = 0.125, p = 0.017), while adding self-compassion (SCS Total) and emotion regulation strategies increased the explained variance to 56.4% (Adjusted R^2^ = 0.564, ΔR^2^ = 0.078, p < 0.001). In the final model for anxiety, pain intensity (VAS) positively predicted anxiety (β = 0.224, p = 0.026), resilience (BRS Total) negatively predicted anxiety (β =  − 0.374, p = 0.006), rumination positively predicted anxiety (β = 0.277, p = 0.007), and catastrophizing positively predicted anxiety (β = 0.250, p = 0.038). These findings highlight the significant roles of self-compassion, resilience, and cognitive emotion regulation strategies in predicting psychological outcomes in FM patients (Please refer to Tables 4, 5 for all regression weights).

**Table 4:** Results of hierarchical multiple linear regression model predicting depression severity in FM group

Model	Dependent variable: BDI	Unstandardized coefficients		Standardized coefficients	t	p	95% CI for B	
		B	SE	Beta			Lower Limit	Upper Limit
Step 1	Age	− 0.109	0.158	− 0.091	− 0.691	0.492	− 0.424	0.206
	Gender	− 1.362	4.407	− 0.041	− 0.309	0.758	− 10.140	7.415
	Level of education	1.869	1.159	0.232	1.612	0.111	− 0.441	4.178
Step 2	Age	− 0.147	0.151	− 0.124	− 0.979	0.331	− 0.447	0.152
	Gender	− 1.578	4.184	− 0.048	− 0.377	0.707	− 9.914	6.757
	Level of education	1.431	1.110	0.177	1.289	0.201	− 0.780	3.642
	VAS	1.738	0.569	0.325	3.056	0.003	0.605	2.871
Step 3	Age	− 0.115	0.097	− 0.097	− 1.191	0.238	− 0.309	0.078
	Gender	− 1.929	2.788	− 0.059	− 0.692	0.491	− 7.486	3.628
	Level of education	0.448	0.762	0.056	0.588	0.558	− 1.071	1.967
	VAS	0.506	0.388	0.095	1.305	0.196	− 0.267	1.279
	SCS Total	− 3.740	1.366	− 0.281	− 2.738	0.008	− 6.462	− 1.017
	TAS-20 Total	0.153	0.083	0.174	1.842	0.070	− 0.013	0.319
	BRS Total	− 4.516	1.043	− 0.426	− 4.329	< 0.001	− 6.596	− 2.437
Step 4	Age	− 0.092	0.095	− 0.077	− 0.971	0.335	− 0.281	0.097
	Gender	− 0.552	2.868	− 0.017	− 0.192	0.848	− 6.283	5.179
	Level of education	0.066	0.830	0.008	0.079	0.937	− 1.592	1.723
	VAS	0.765	0.396	0.143	1.931	0.058	− 0.027	1.556
	SCS Total	− 1.010	1.873	− 0.076	− 0.539	0.592	− 4.753	2.734
	TAS-20 Total	0.115	0.084	0.131	1.377	0.173	− 0.052	0.283
	BRS Total	− 4.116	1.055	− 0.388	− 3.900	< 0.001	− 6.225	− 2.007
	Self-blame	0.198	0.255	0.072	0.776	0.440	− 0.311	0.707
	Acceptance	− 0.407	0.216	− 0.147	− 1.883	0.064	− 0.839	0.025
	Rumination	0.624	0.233	0.201	2.682	0.009	0.159	1.089
	Positive Refocus	− 0.244	0.335	− 0.088	− 0.728	0.470	− 0.914	0.426
	Refocus on Planning	0.449	0.332	0.173	1.352	0.181	− 0.215	1.112
	Positive Reappraisal	− 0.932	0.396	− 0.335	− 2.355	0.022	− 1.722	− 0.141
	Diminishing the value of the event	0.181	0.260	0.058	0.697	0.488	− 0.339	0.701
	Catastrophizing	0.215	0.216	0.088	0.995	0.324	− 0.217	0.648
	Blaming Others	− 0.117	0.201	− 0.049	− 0.583	0.562	− 0.518	0.284

*BDI* Beck Depression Inventory, *BAI* Beck Anxiety Inventory, *SCS* Self-Compassion Scale, *TAS-20* Toronto Alexithymia Scale, *VAS* Visual Analog Scale, *BRS* Brief Resilience Scale, *SE* Standart Error, *B* Unstandardized regression weight

**Table 5:** Results of hierarchical multiple linear regression model predicting anxiety severity in FM group

Model	Dependent variable: BAI	Unstandardized Coefficients		Standardized Coefficients	t	p	95% CI for B	
		B	SE	Beta			Lower Limit	Upper Limit
Step 1	Age	0.140	0.162	0.112	0.867	0.389	− 0.182	0.463
	Gender	− 14.582	4.520	− 0.420	− 3.226	0.002	− 23.585	− 5.579
	Level of education	2.268	1.189	0.267	1.907	0.060	− 0.101	4.636
Step 2	Age	0.097	0.152	0.077	0.636	0.526	− 0.206	0.400
	Gender	− 14.829	4.229	− 0.427	− 3.507	0.001	− 23.253	− 6.405
	Level of education	1.769	1.122	0.208	1.577	0.119	− 0.466	4.003
	VAS	1.981	0.575	0.351	3.446	0.001	0.836	3.125
Step 3	Age	0.128	0.132	0.102	0.968	0.336	− 0.136	0.392
	Gender	− 14.757	3.810	− 0.425	− 3.873	< 0.001	− 22.352	− 7.162
	Level of education	0.926	1.041	0.109	0.889	0.377	− 1.150	3.001
	VAS	1,096	0.530	0.194	2.068	0.042	0.039	2.152
	SCS Total	− 2.170	1.867	− 0.155	− 1.162	0.249	− 5.891	1.552
	TAS-20 Total	0.060	0.114	0.064	0.523	0.603	− 0.167	0.286
	BRS Total	− 3.890	1.426	− 0.348	− 2.728	0.008	− 6.732	− 1.047
Step 4	Age	0.159	0.133	0.126	1.195	0.236	− 0.107	0.425
	Gender	− 12.778	4.034	− 0.368	− 3.167	0.002	− 20.840	− 4.716
	Level of education	0.712	1.167	0.084	0.610	0.544	− 1.620	3.044
	VAS	1.267	0.557	0.224	2.273	0.026	0.153	2.380
	SCS Total	− 1.064	2.635	− 0.076	− 0.404	0.688	− 6.330	4.202
	TAS-20 Total	0.070	0.118	0.075	0.596	0.553	− 0.165	0.306
	BRS Total	− 4.182	1.485	− 0.374	− 2.817	0.006	− 7.149	− 1.216
	Self-blame	− 0.435	0.359	− 0.151	− 1.212	0.230	− 1.151	0.282
	Acceptance	− 0.394	0.304	− 0.135	− 1.295	0.200	− 1.002	0.214
	Rumination	0.908	0.327	0.277	2.773	0.007	0.254	1.562
	Positive refocus	0.354	0.471	0.121	0.751	0.456	− 0.588	1.296
	Refocus on planning	0.091	0.467	0.033	0.196	0.845	− 0.842	1.025
	Positive Reappraisal	− 0.488	0.556	− 0.166	− 0.878	0.384	− 1.600	0.624
	Diminishing the value of the event	0.192	0.366	0.058	0.526	0.601	− 0.539	0.924
	Catastrophizing	0.646	0.304	0.250	2.120	0.038	0.037	1.254
	Blaming others	− 0.269	0.282	− 0.106	− 0.953	0.344	− 0.833	0.295

*BDI* Beck Depression Inventory, *BAI* Beck Anxiety Inventory, *SCS* Self-Compassion Scale, *TAS-20* Toronto Alexithymia Scale, *VAS* Visual Analog Scale, *BRS* Brief Resilience Scale, *SE* Standart Error, *B* Unstandardized regression weight

### Mediation analysis

**Fig. 1:**
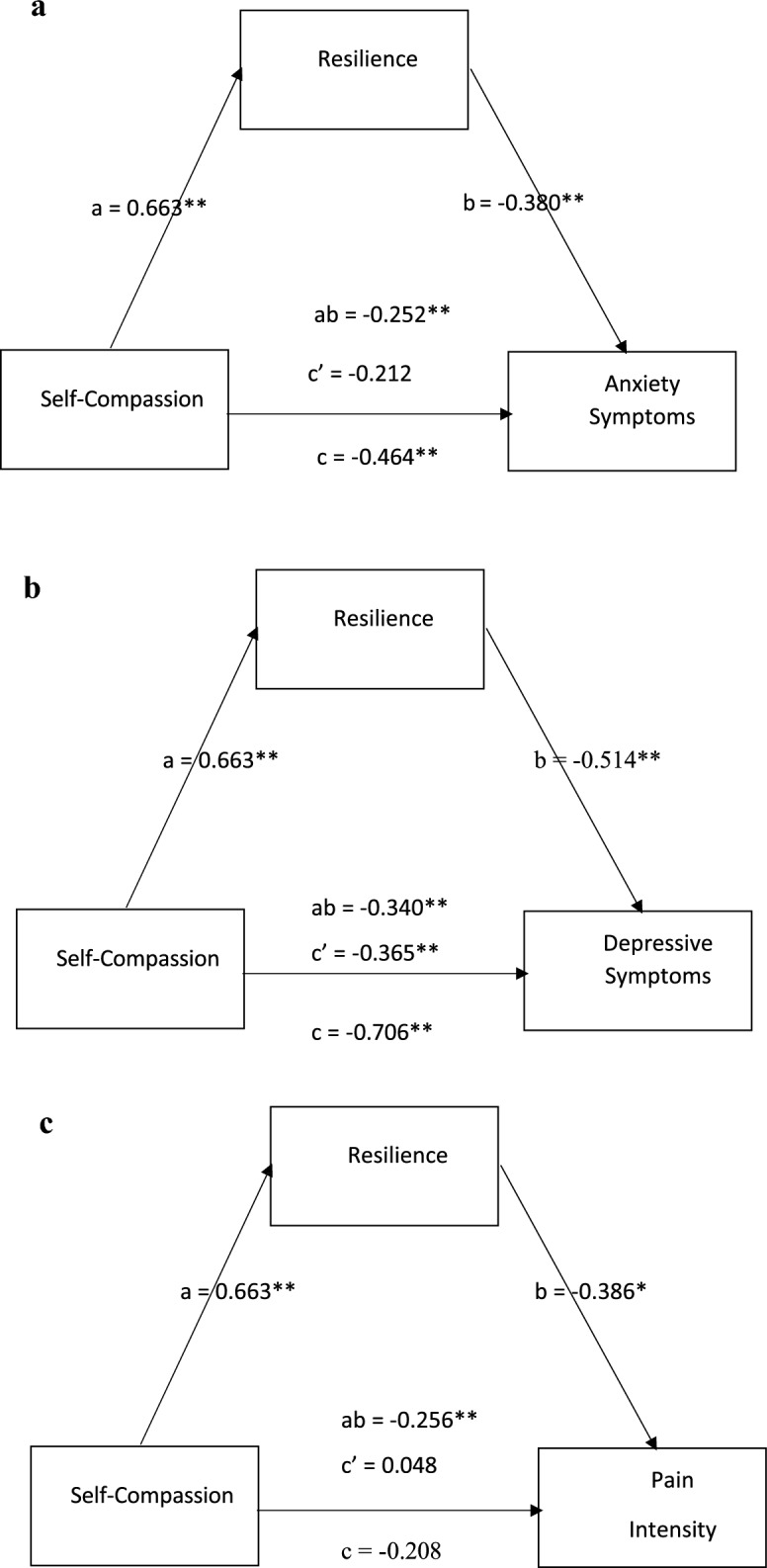
**a** The mediation model of resilience in the relationship between self-compassion and anxiety symptoms. Path coefficients for c total effect (self-compassion related to anxiety symptoms), c'direct effect (self-compassion related to anxiety symptoms accounting for resilience), and ab indirect effect (self-compassion related to anxiety symptoms through resilience) are presented with significance denoted as **p < 0.001. **b** The mediation model of resilience in the relationship between self-compassion and depressive symptoms. Path coefficients for c total effect (self-compassion related to depressive symptoms), c'direct effect (self-compassion related to depressive symptoms accounting for resilience), and ab indirect effect (self-compassion related to depressive symptoms through resilience) are presented with significance levels denoted as **p < 0.001. **c** The mediation model of resilience in the relationship between self-compassion and pain intensity. Path coefficients for c total effect (self-compassion related to pain intensity), c'direct effect (self-compassion related to pain intensity accounting for resilience), and ab indirect effect (self-compassion related to pain intensity through resilience) are presented with significance levels denoted as *p < 0.01, **p < 0.001

In a simple mediation model, resillience significantly mediated the relationship between self-compassion and health outcomes (anxiety, depression, and pain intensity) with varying significance. For anxiety symptoms, self-compassion was significantly related to resilience (a = 0.663, p < 0.001), and resilience was significantly associated with reduced anxiety symptoms (b =  − 0.380, p < 0.001). However, the direct effect of self-compassion on anxiety symptoms was not significant after accounting for resilience (c'= − 0.212, p = 0.129), suggesting significant full mediation in this case. The indirect effect (ab =  − 0.252, BCa 95% CI: [− 0.39, − 0.11]) was significant, confirming an indirect effect of self-compassion on anxiety through resilience. For depressive symptoms, self-compassion was significantly related to resilience (a = 0.663, p < 0.001), and resilience significantly reduced depressive symptoms (b = −0.514, p < 0.001). The direct effect of self-compassion on depression decreased after accounting for resilience (c'= − 0.366, p < 0.001), confirming significant partial mediation. The indirect effect (ab =  − 0.340, BCa 95% CI: [− 0.32, − 0.09]) was also significant. For pain intensity, self-compassion was significantly related to resilience (a = 0.663, p < 0.001), and resilience was significantly associated with reduced pain intensity (b =  − 0.386, p < 0.01). However, after accounting for resilience, the direct effect of self-compassion on pain intensity was not significant (c'= 0.048, p = 0.639), and the total effect was also non-significant, suggesting no significant mediation in this case. The indirect effect [ab =  − 0.256, BCa 95% CI: (− 0.33, − 0.10)] was significant, although resillience did not significantly mediate the relationship between self compassion and pain intensity. In summary, resilience was found to partially mediate the relationship between self-compassion and depression, indicating that self-compassion influenced depression both directly and indirectly through resilience. In contrast, resilience fully mediated the relationship between self-compassion and anxiety, suggesting that the effect of self-compassion on this outcome was entirely channeled through resilience. All mediation models are demonstrated in Fig. 1.

## Discussion

This study examined key psychological factors associated with FM, specifically self-compassion, cognitive emotion regulation, and their relationships with depression, anxiety, and resilience. Compared to healthy controls, individuals with FM reported significantly lower levels of self-compassion and a greater reliance on maladaptive emotion regulation strategies, such as rumination and catastrophizing. Self-compassion was inversely associated with depression and anxiety, emphasizing its protective role in psychological well-being. In contrast, maladaptive emotion regulation strategies were positively associated with both depression and anxiety, highlighting their adverse impact on mental health. Notably, resilience emerged as a mediating factor in these associations: higher levels of self-compassion predicted greater resilience, which, in turn, was linked to reduced symptoms of depression and anxiety. These findings suggest that psychological interventions targeting self-compassion and adaptive emotion regulation may offer meaningful benefits for improving emotional well-being and overall quality of life in individuals with FM.

These findings underscore the central role of psychological factors in the management of FM, particularly the detrimental impact of maladaptive emotion regulation strategies such as rumination and catastrophizing. These strategies not only exacerbate psychological distress but may also intensify both emotional and physical symptomatology, potentially creating a self-reinforcing cycle of pain and negative affect. This is consistent with prior research highlighting emotion regulation as a key determinant in the psychological experience of chronic pain [15, 16]. Our findings extend this literature by suggesting that these cognitive-emotional tendencies are not merely correlates of distress but may function through resilience-related mechanisms. Notably, the association between rumination and sustained psychological burden supports earlier findings identifying rumination as a central factor in the persistence of both pain and affective symptoms in FM [34, 35]. While previous studies have predominantly described these associations, our study adds to the literature by modeling resilience as a mediator, thereby offering a more process-oriented understanding of the emotional adaptation mechanisms in FM. Additionally, accumulating neurobiological evidence—such as that related to central sensitization and small fiber neuropathy (SFN)—underscores the interplay between emotional and sensory dysregulation in FM, further supporting the need for integrated biopsychosocial interventions [4]. Conversely, self-compassion emerged as a protective factor against depression and anxiety, consistent with earlier findings that highlight its buffering role in psychological adjustment [9, 36]. Prior studies have shown that self-compassion facilitates adaptive coping through enhanced mindfulness, self-kindness, and a sense of shared humanity [18]. These attributes are especially pertinent for individuals with FM, who frequently experience pain-related self-criticism and emotional isolation. Recent research, including work in chronic health populations such as FM, has confirmed that higher self-compassion is associated with reduced psychological distress and improved emotion regulation capacity [37]. Our findings support and expand upon this work by showing that the beneficial effects of self-compassion may be partly mediated by increased resilience. However, not all studies have found self-compassion to be consistently predictive of improved physical outcomes. Some research suggests that self-compassion may not significantly influence pain severity or disability, indicating that its primary benefits may lie in alleviating emotional—rather than somatic—distress [38, 39]. Our results align with this interpretation, reinforcing the notion that while self-compassion can serve as a critical psychological buffer in FM, its influence on physical symptomatology may be more limited. To contextualize these findings within the broader literature, it is useful to consider prior work that has examined similar psychological constructs in FM. Previous studies have consistently highlighted the role of psychological mechanisms in the experience and management of fibromyalgia symptoms. For instance, personality-related traits have also been shown to play a significant role; Gökcen et al. (2022) reported that Type D personality is highly prevalent among FM patients and is closely linked to increased anxiety, depression, and reduced self-esteem [40]. Furthermore, intervention studies have suggested that cultivating psychological flexibility and awareness may yield long-term benefits. Mayer et al. [41] observed that participants in a structured mindfulness-based program experienced sustained reductions in fatigue and emotional distress [41]. Similarly, Zangi et al. [42] demonstrated that a mindfulness-based group program followed by physical activity counseling led to moderate improvements in FM severity over a two-year follow-up period [42]. Despite these advancements, existing studies often examine isolated psychological factors. In contrast, the present study integrates self-compassion, emotion regulation, and resilience within a single predictive model, offering a more comprehensive perspective on psychological well-being in FM. This multidimensional framework may better inform targeted interventions that address the interplay between these adaptive traits.

The findings from this study underscore the central role of resilience in managing psychological distress in FM patients. Specifically, the positive association between resilience and self-compassion suggests that individuals with higher levels of self-compassion are better equipped to cope with the challenges of FM. This resilience, in turn, plays a critical role in buffering the negative effects of depression and anxiety. These results are consistent with prior research showing that resilience helps individuals with chronic pain conditions, such as FM, to cope more effectively with emotional distress and physical symptoms [43]. Resilience promotes adaptive coping, reduces psychological vulnerability, and contributes to better health outcomes [44]. Furthermore, research by Zautra et al. found that psychological resilience serves as a protective factor against the negative impact of pain and depression in women with fibromyalgia, supporting the notion that resilience can buffer the emotional toll of chronic illness [45]. These findings reinforce the idea that fostering self-compassion not only alleviates psychological distress but also enhances resilience, which is essential for improving both emotional well-being and the management of the psychological and physical challenges inherent in FM.

The regression analyses revealed that both self-compassion and maladaptive emotion regulation strategies are significant predictors of depression and anxiety in individuals with FM. Specifically, self-compassion emerged as a strong negative predictor, suggesting that individuals with higher levels of self-compassion experience less psychological distress. This finding aligns with prior research demonstrating that self-compassion is associated with improved mental health outcomes across chronic conditions, including FM [46]. However, some authors have criticized the overreliance on self-report measures in self-compassion research, arguing that current instruments may not fully capture the protective function of self-compassion across different populations [47]. In contrast, rumination—a maladaptive cognitive coping strategy—was a significant positive predictor of both depression and anxiety, consistent with findings that link rumination to the persistence of emotional symptoms and the amplification of pain perception in chronic pain conditions [48, 49]. This supports theoretical models such as the fear-avoidance model, which posits that catastrophic thinking and rumination perpetuate a cycle of heightened pain and psychological distress [50, 51]. Hierarchical multiple regression models confirmed the hypothesized relationships between self-compassion, emotion regulation strategies, resilience, and psychological outcomes. These models explained 75.5% of the variance in depression and anxiety scores, emphasizing the substantial influence of psychological variables in FM. This supports prior research demonstrating that self-compassion and adaptive emotional regulation strategies serve as protective psychological mechanisms in managing chronic pain [12, 52]. Moreover, resilience was identified as a significant mediator, amplifying the beneficial impact of self-compassion on depression and anxiety—reinforcing its role as a buffer against emotional distress in individuals living with chronic conditions [43, 53]. These results further corroborate findings from resilience literature, which emphasizes adaptive coping and psychological flexibility as essential components of pain management and emotional well-being [54]. Additionally, maladaptive strategies such as rumination and catastrophizing significantly contributed to emotional distress in FM, reaffirming their deleterious impact on psychological functioning as emphasized in earlier studies [12, 14]. The robustness of the regression models lends support to the theoretical proposition that adaptive psychological constructs—such as self-compassion, resilience, and emotion regulation—can meaningfully reduce the emotional burden of FM. This is in line with Neff and Germer’s framework [9], which identifies self-compassion as a pivotal factor in enhancing emotional regulation and psychological flexibility. Taken together, these findings call for an integrative treatment approach that addresses both psychological and somatic dimensions of FM. Future research should employ longitudinal and experimental designs to clarify causal pathways and explore additional psychosocial influences—such as perceived stigma, social support, and illness beliefs—on the mental health outcomes of individuals with FM (see Fig. 1).

Our findings indicate that self-compassion had both a direct effect and an indirect effect  on depressive symptoms through resillience, aligning with previous research suggesting that individuals with higher self-compassion are better equipped to cope with chronic conditions such as FM [55, 56]. Self-compassion was associated with a reduction in depressive symptoms, supporting the idea that a kind and accepting attitude toward oneself serves as a buffer against stress and emotional distress [18, 57]. While resilience was positively related to self-compassion, self-compassion was not directly related to anxiety or pain intensity in our model, suggesting that its impact may be more pronounced for depressive symptomatology. This finding resonates with previous studies showing that self-compassion is particularly effective in alleviating emotional distress and fostering adaptive coping strategies such as acceptance and cognitive reframing [55, 58]. However, not all studies have found uniform effects. For instance, Costa and Pinto-Gouveia [39] reported that while self-compassion reduced emotional suffering, it did not significantly predict pain intensity or functional disability, indicating that its benefits may be limited primarily to the affective dimension of FM. Similarly, individual differences in psychological flexibility, internalized stigma, or cultural attitudes toward self-kindness may moderate the extent to which self-compassion exerts its protective effects [59]. Viewed differently, the mediating role of resilience highlights its importance as a mechanism through which self-compassion influences well-being.  Prior work has shown that resilience serves as a protective factor against both anxiety and pain-related distress in individuals with chronic pain conditions [54, 60]. Moreover, self-compassion has been found to reduce threat-based emotional reactivity and facilitate self-soothing processes [18], which likely contribute to resilience development. These discrepancies highlight the need for further investigation into the boundary conditions under which self-compassion operates most effectively. Nonetheless, our findings underscore self-compassion as a crucial psychological resource for individuals with FM, helping them reframe their experiences and adopt healthier coping mechanisms, which can ultimately enhance mental health–related quality of life [61, 62]. Given the multifaceted nature of FM, comprehensive treatment approaches are essential. Interventions that promote self-compassion, resilience, and adaptive emotion regulation should be prioritized as part of integrative care strategies to reduce psychological distress and improve overall disease management.

This study has several limitations. First, the reliance on a clinical sample of FM patients and healthy controls may limit generalizability, as it may not capture the full heterogeneity of symptom severity and psychological complexity observed in broader or primary care populations. FM patients from rheumatology clinics often differ in psychosocial functioning from those in othersettings, potentially introducing selection bias [59]. Additionally, the absence of detailed information on comorbid chronic pain conditions may confound interpretations, as overlapping symptomatology could affect psychological outcomes. Second, the cross-sectional design precludes causal inferences regarding the relationships between self-compassion, emotion regulation, resilience, and psychological distress. Longitudinal studies are needed to clarify temporal and directional patterns [13]. Third, the exclusive use of self-report instruments may introduce biases such as social desirability and recall errors [63]. Future studies should consider multimethod approaches, including clinician-rated or physiological measures. Despite these limitations, this study contributes meaningfully by addressing the often-overlooked psychological dimensions of FM. Unlike prior research that has focused predominantly on physical symptoms, this study underscores the relevance of self-compassion and emotion regulation in promoting resilience and reducing distress. These findings support a biopsychosocial model of FM care and advocate for incorporating psychological interventions—such as compassion-focused and emotion regulation-based therapies—into multidisciplinary treatment plans [64].

In conclusion, this study highlights the importance of self-compassion and adaptive emotion regulation strategies in alleviating psychological distress in individuals with FM. Regression analyses identified self-compassion and emotion regulation as strong predictors of depression and anxiety, with resilience emerging as a crucial mediator, particularly in the relationship between self-compassion and depressive symptoms. However, no significant direct effect was observed for anxiety or pain intensity. A  noteworthy indirect effect was found only for anxiety, while no significant mediation was observed for pain intensity. These differential patterns suggest that while self-compassion may buffer depression through multiple mechanisms—including emotional awareness, cognitive reframing, or behavioral coping—its effect on anxiety and pain may depend more strongly on the individual's capacity to bounce back from stress. These findings suggest that fostering self-compassion and adaptive emotion regulation can enhance resilience and alleviate depressive symptoms. Integrating interventions that target these psychological factors into FM management could significantly improve emotional well-being and reduce psychological distress. A holistic approach that addresses both the physical and emotional challenges of FM is essential for effective treatment [65, 66]. Future interventions should focus on developing self-compassion, enhancing emotion regulation skills, and strengthening resilience to mitigate psychological suffering, improve mental health outcomes, and enhance the overall quality of life for individuals with FM.

## Data Availability

The datasets and materials during the present study are available from the corresponding author on request.

## Declarations

### Disclaimer

No part of this manuscript, including the text and graphics, has been copied or published elsewhere in whole or in part in any language. All content is original and has not been previously submitted or published in any form, including conference abstracts or proceedings. AI tools were not used for language editing and writing.
